# Intravitreal vascular endothelial growth factor and connective tissue growth factor levels and risk factors associated with vitreous hemorrhage in proliferative diabetic retinopathy after pan-retinal laser photocoagulation: a cross-sectional study

**DOI:** 10.1186/s40942-025-00784-0

**Published:** 2026-01-02

**Authors:** Zechen Liu, Chang Shu, Xiaorong Li, Jindong Han

**Affiliations:** Tianjin Key Laboratory of Retinal Functions and Diseases, Tianjin Branch of National Clinical Research Center for Ocular Disease, Eye Institute and School of Optometry, Tianjin Medical University Eye Hospital, Tianjin, China

**Keywords:** Vascular endothelial growth factor, Connective tissue growth factor, Pan-retinal photocoagulation, Vitreous humor, Proliferative diabetic retinopathy

## Abstract

**Background:**

The study aimed to investigate the changes of vascular endothelial growth factor (VEGF) and connective tissue growth factor (CTGF) concentrations in proliferative diabetic retinopathy (PDR) combined with vitreous hemorrhage (VH) patients after panretinal photocoagulation (PRP), and to explore the risk factors associated with the occurrence of VH.

**Methods:**

This cross-sectional study included 46 eyes surgically treated with pars plana vitrectomy (PPV), divided into three groups: PRP group (18 eyes) – PDR combined with VH patients with previous PRP; no-PRP group (18 eyes) – PDR combined with VH patients without previous PRP; and control group (10 eyes) – PPV performed for idiopathic macular hole (IMH) without diabetes mellitus. Both PRP and no-PRP groups underwent surgery due to non-clearing VH. Vitreous samples were obtained during PPV, then VEGF and CTGF concentrations were determined by enzyme-linked immunosorbent assay (ELISA).

**Results:**

Intravitreal VEGF levels were significantly lower in the PRP group (1968.10[1423.96-2341.68] pg/mL) than in the no-PRP group (2815.71[2440.70-3395.52] pg/mL) (*P* = 0.015), but CTGF levels showed no statistical difference. The CTGF/VEGF ratio was significantly higher in the PRP group (0.27[0.13–0.34]) than in the no-PRP group (0.13[0.11–0.18]) (*P* = 0.002). Multiple linear regression analysis revealed that PRP treatment was independently associated with lower VEGF levels (*P* < 0.001) and a higher CTGF/VEGF ratio (*P* = 0.004). A positive correlation was observed between vitreous VEGF and CTGF levels in the no-PRP group (*r* = 0.486, *P* = 0.041), whereas no correlation was observed in the PRP group.

**Conclusion:**

PRP treatment was associated with an alteration in the relationship between VEGF and CTGF levels in PDR patients, characterized by an upregulation of the CTGF/VEGF ratio and a pro-fibrotic shift. Traction from microfibrous vascular membranes and the vitreous may represent key mechanisms contributing to VH in PDR patients after PRP treatment.

**Trial registration:**

Trial registration number ChiCTR2400088011. Date of registration July 23, 2024.

## Background

Proliferative diabetic retinopathy (PDR), a leading cause of vision impairment in the elderly, is characterized by retinal neovascularization driven by chronic retinal hypoxia. The hypoxic environment stimulates the overproduction of pro-angiogenic factors, promoting endothelial cell migration and proliferation and, eventually, the formation of abnormal new vessels [1]. Similar to a wound healing response, with the influx of inflammatory cells and contraction of myofibroblasts, the new vessels may develop into fibrous vascular tissue, ultimately causing vitreous hemorrhage (VH) and tractional retinal detachment [2].

Vascular endothelial growth factor (VEGF) is the predominant pro-angiogenic cytokine and plays an important role in inducing blood-retinal barrier disruption, increased vascular leakage and retinal angiogenesis [3]. Connective tissue growth factor (CTGF) is a secreted matricellular protein with pro-fibrotic activity that is upregulated during tissue remodeling. It is involved in retinal capillary basement membrane thickening and pericyte loss, and facilitates the transition of PDR from neovascularization to the fibrotic stage [4, 5]. VEGF induces CTGF expression, which in turn inhibits VEGF-induced angiogenesis by forming CTGF-VEGF binding proteins [6, 7]. Furthermore, both vitreous CTGF levels and the CTGF/VEGF ratio are strongly associated with fibrotic progression in PDR, with the latter being the strongest predictor of degree of fibrosis and the angio-fibrotic switch [8–10]. In addition, CTGF may also promote ocular angiogenesis under specific conditions, but the evidence is inconclusive [11, 12].

Panretinal photocoagulation (PRP) remains the standard treatment for effectively preventing visual impairment in patients with severe NPDR and PDR [13]. By reducing retinal oxygen demand, PRP reduces VEGF levels in the vitreous cavity and promotes regression of neovascularization [14]. Although previous studies have suggested that PRP may induce a transient angio-fibrotic switch [9], data on the subsequent changes in CTGF levels and the CTGF/VEGF ratio remain limited. In addition, approximately one-third of PDR patients still develop VH and require pars plana vitrectomy (PPV) following PRP [15], for reasons that remain incompletely understood. This study aimed to investigate changes in VEGF and CTGF concentrations in PDR patients after PRP treatment, and further explore the pathogenic mechanisms and risk factors underlying the occurrence of VH.

## Methods

### Study design and setting

**Fig. 1:**
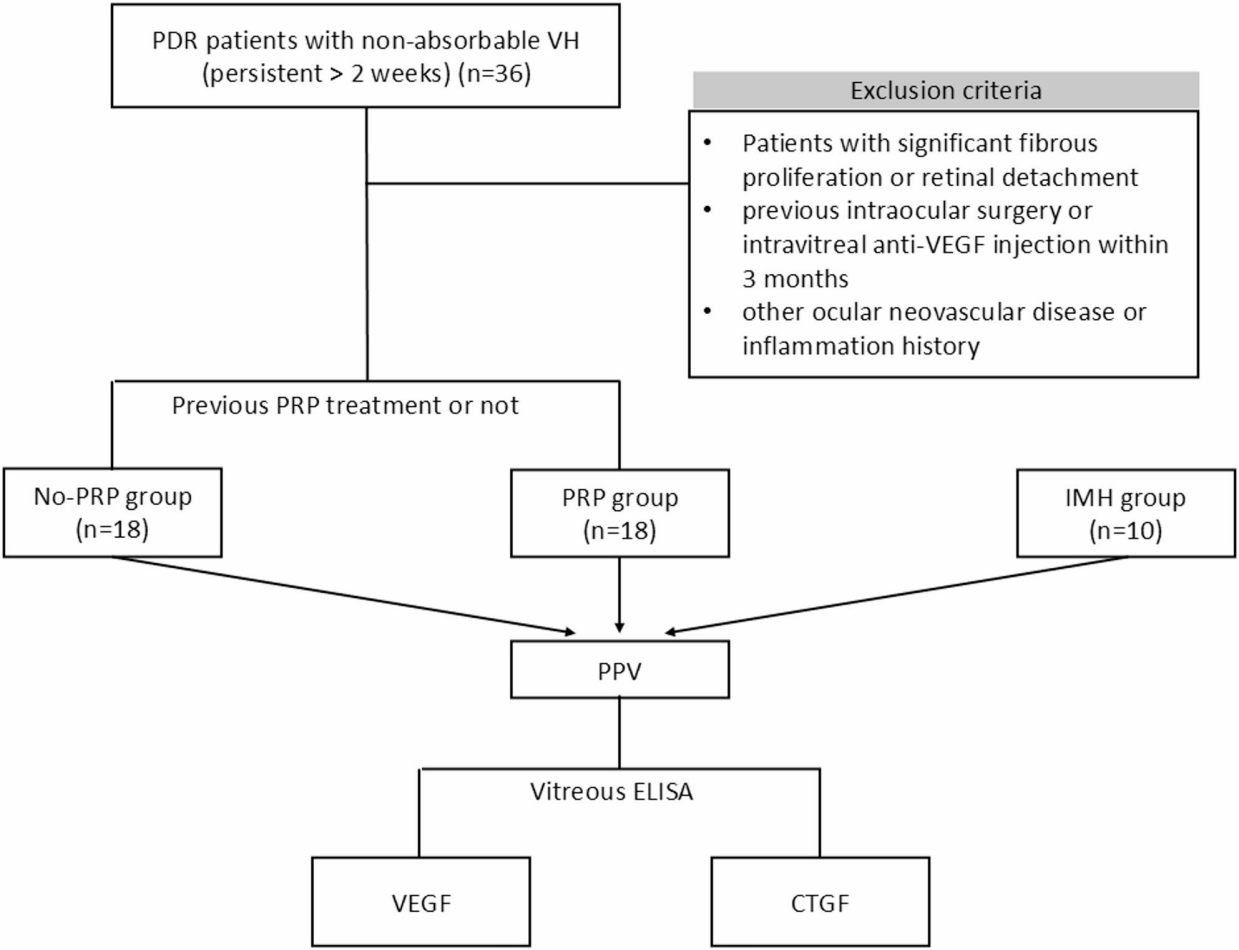
The flow chart

This cross-sectional study was conducted at the Tianjin Medical University Eye Hospital. We performed an a priori sample size calculation using PASS 15 software. Based on the primary outcome of vitreous VEGF levels from pilot data and literature [16], a total of 24 participants (12 per group) were required to detect a large effect size between the study groups with 90% power at a two-sided alpha level of 0.05. We ultimately enrolled 36 PDR eyes treated with PPV for non-clearing VH from August to October 2024 to enhance the robustness of the study. These included 18 eyes previously treated with PRP (PRP group) and 18 eyes that had not previously received PRP treatment (no-PRP group). Ten non-diabetic eyes with idiopathic macular hole (IMH) were included as a control group during the same period and were not part of the power calculation (Fig. 1).

The inclusion criteria for PDR eyes were: (1) persistent VH (> 2 weeks) affecting vision and requiring PPV; and (2) for the PRP group specifically, a history of at least three to four previous PRP sessions. Exclusion criteria included: (1) significant fibrous proliferation or tractional retinal detachment; (2) any history of intraocular surgery (except for uncomplicated cataract surgery) or intravitreal anti-VEGF injections within the preceding 3 months; and (3) a history of other ocular neovascular diseases or inflammation.

### Data collection

Baseline information included age, gender, best-corrected visual acuity (BCVA), systolic and diastolic pressure, duration of diabetes mellitus (DM), and type of posterior vitreous detachment (PVD). Laboratory parameters, including leukocyte, erythrocyte, HbA1c, cholesterol, and triglycerides were recorded. BCVA was measured using a standard logarithmic visual acuity chart and converted to a logarithm of the minimum angle of resolution (logMAR), with logMAR values assigned as 1.98 for counting finger and 2.28 for hand motion. Slit-lamp biomicroscopy examination, B-scan ultrasonography (US), and fundus photography were performed before surgery. The classification of PVD was primarily determined by preoperative US and confirmed by intraoperative observation (preoperative optical coherence tomography was usually not feasible due to VH). To ensure reliability, all US images were independently evaluated by two ophthalmologists: first by an attending physician and then by a senior vitreoretinal specialist. Any discrepancies in interpretation were resolved by the senior specialist. Intraoperative confirmation was performed by the same senior ophthalmologist using intravitreal injection of triamcinolone acetonide 0.5 mg.

**Fig. 2:**
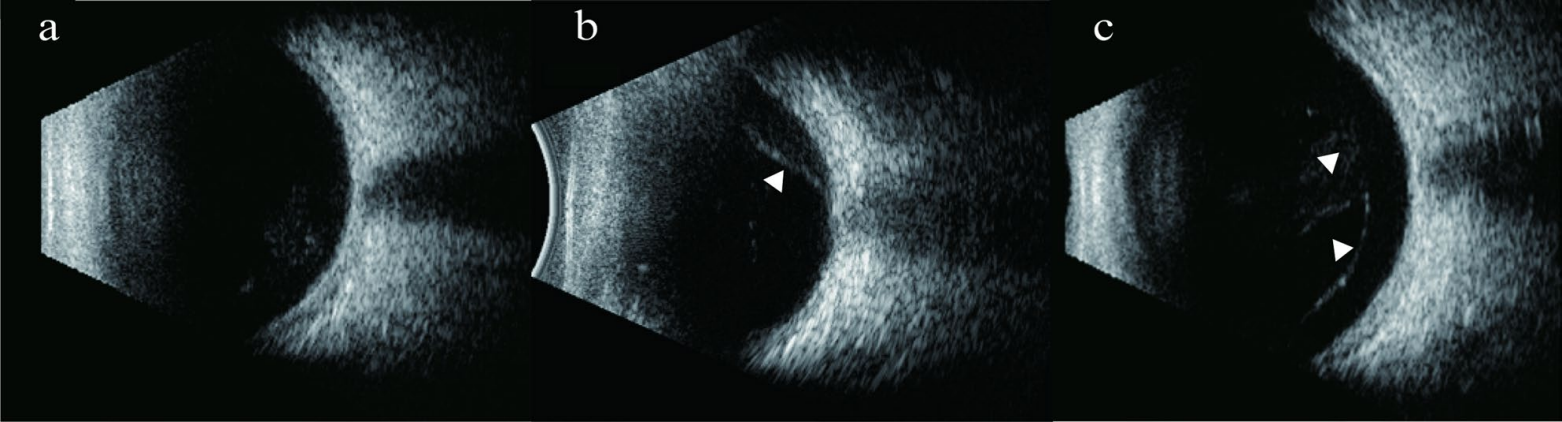
Classification of PVD based on US. (**a**) No PVD: no hyperechoic membrane was observed over retina. (**b**) Partial PVD: a hyperechoic membrane partially detached from the posterior retina was observed(arrowhead). (**c**) Complete PVD: a completely detached hyperechoic membrane was observed over the posterior retina (arrowheads)

Depending on the presence of a complete separation between posterior vitreous cortex (PVC) and inner limiting membrane (ILM), PVD is classified as: (1) No PVD: no PVD in the macular and optic disc areas; (2) Partial PVD: PVD in or around the macular or optic disc area; and (3) Complete PVD: PVD is in both the macular and optic disc areas [17]. All US images were centered on the optic disc shadow. No PVD was defined in US images if no hyperechoic membrane was observed over the retina. Partial PVD was defined as a hyperechoic membrane detached from the posterior retina but still partially adherent. Complete PVD was defined as complete detachment of hyperechoic membrane from the posterior retina (Fig. 2).

### Sample collection and ELISA

Undiluted vitreous samples (0.5mL) were obtained at the beginning of standard three-port PPV by aspirating into a 1mL sterile syringe before opening the infusion port. Samples were transferred to sterile Eppendorf tubes, placed on ice, and then immediately stored at -80℃ until analysis. After thawing, the samples were centrifuged at 1000xg for 20 min at 4 °C, and the precipitate-free supernatant was collected. VEGF and CTGF concentrations were measured by enzyme-linked immunosorbent assay (ELISA) (Jiangsu Jingmei Biotechnology Co., Ltd., China).

### Statistical analysis

Statistical analyses were performed using Statistical Package for Social Sciences V.27.0 (SPSS V.27.0, USA). Values were expressed as median and 25th -75th quartiles. Continuous parametric data were analyzed by Kruskal-Wallis or Mann-Whitney U test and the post hoc Bonferroni correction was used. The Chi-Squared or Fisher’s exact tests were used to compare baseline differences in categorical data. Multiple linear regression analyses were employed to assess the independent association of PRP treatment with vitreous cytokine levels. The selection of covariates was primarily based on their established clinical relevance to diabetic retinopathy, including diabetes duration, HbA1c, and PVD status, rather than solely on statistical significance in univariate analyses. Model assumptions of linearity, homoscedasticity, and normality of residuals were verified and acceptable. Results are presented as unstandardized beta (B) coefficients with 95% confidence intervals (CI). Spearman rank correlation was performed to explore correlations between CTGF and VEGF levels. Receiver operating characteristic (ROC) curves and the corresponding area under the curve (AUC) were used to identify the optimal threshold for vitreous factor concentrations in PDR patients with or without previous PRP. Statistical significance was indicated by *P* < 0.05. A post hoc power analysis for the subgroup comparison was performed using G*Power software (version 3.1.9.7).

## Results

### Patient characteristics

The baseline characteristics of patients across the three groups are summarized in Table 1. No significant differences were observed among the groups in terms of age, systolic, and diastolic blood pressure. There were no statistically significant differences in gender and duration of DM between the PRP and no-PRP groups, but 90% of patients in the control group were female, which differed significantly from the PRP group (*P* = 0.017). Baseline BCVA was lowest in the no-PRP group at logMAR1.98 (1.523–2.28), which was significantly different from the PRP group logMAR0.398 (0.301–1.301) (*P* < 0.001). With regard to PVD types, significant differences were observed between the two experimental groups (*P* = 0.023). Overall, no PVD was present in 18 eyes (50%), partial PVD in 13 eyes (36.1%), and complete PVD in only 5 eyes (13.9%). No PVD was observed in the majority of eyes in the PRP group, whereas about half of eyes in the no-PRP group had partial PVD.

Laboratory parameters for the three groups are summarized in Table 2. No significant differences were observed in leukocytes, erythrocytes, and triglycerides among the groups. Regarding HbA1c and cholesterol, there was no significant difference between the PRP and no-PRP groups, but was statistically different from the control group (*P* = 0.006 and *P* = 0.004, *P* = 0.03 and *P* = 0.01, respectively).

**Table 1:** Baseline characteristics

	PRP group (*n* = 18)	No-PRP group (*n* = 18)	Control group (*n* = 10)	*P* value
Age	57.00(50.50–62.50)	56.50(50.50–63.50)	63.50(60.00-67.75)	0.054^^^
Female gender, no. (%)	7(38.9)	8(44.4)	9(90)	**0.017** ^**#**^
BCVA (logMAR)	0.398(0.301–1.301)	1.98(1.523–2.28)	-	**< 0.001** ^**~**^
Duration of DM, years	15(7.50-20.25) 10(3-15.5)	-		0.318^~^
Systolic pressure(mmHg)	146(137–160)	153(139–170)	132(131–148)	0.129^^^
Diastolic pressure(mmHg)	81(77–89)	93(80–100)	79(76–93)	0.174^^^
PVD type				
No PVD, no (%)	13(72.2)	5(27.8)	-	
Partial PVD, no (%)	4(22.2)	9(50)		**0.023** ^**#**^
Complete PVD, no (%)	1(5.6)	4(22.2)	-	

BCVA, best corrected visual acuity; logMAR, logarithm of minimal angle resolution; DM, diabetes mellitus; ^^^Kruskal–Wallis test. ^#^Chi-Squared test or Fisher’s exact test. ^~^Mann-Whitney U test

**Table 2:** Patients’ laboratory parameters

	PRP group (*n* = 18)	No-PRP group (*n* = 18)	Control group (*n* = 10)	*P* value
Leukocyte number/ L	7.68(6.22–8.62)	6.37(4.21–8.91)	6.13(5.15–6.69)	0.073^^^
Erythrocyte number/ L	4.51(4.29–4.97)	4.47(3.91–4.98)	4.52(4.16–4.82)	0.811^^^
HbA1c (%)	7.25(6.40–7.70)	7.30(6.50–8.90)	5.40(4.95–7.80)	**0.002** ^**^**^
Cholesterol (mmol/L)	4.20(3.73-5.00)	4.05(3.03–5.73)	5.40(4.95–7.80)	**0.009** ^**^**^
Triglyceride (mmol/L)	1.36(0.91–2.06)	1.32(1.06–1.68)	1.14(0.84–1.98)	0.834^^^

^^^Kruskal–Wallis test with post-hoc Bonferroni correction

### VEGF and CTGF concentrations in vitreous samples

**Fig. 3:**
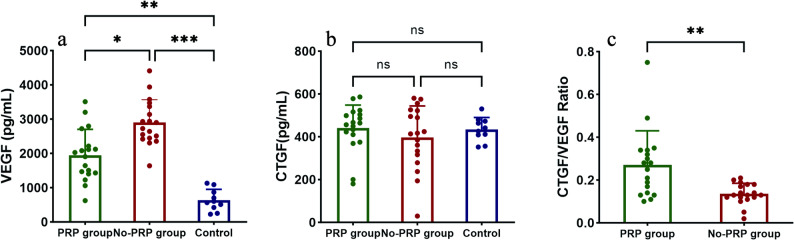
Vitreous concentrations of vascular endothelial growth factor (VEGF) (**a**), connective tissue growth factor (CTGF) (**b**) and CTGF/VEGF ratio (**c**) in the three groups (ns: *P* > 0.05; *P ≤ 0.05; **P ≤ 0.01; ***P ≤ 0.001)

Of the three groups, vitreous VEGF concentration was significantly lower in the PRP group (1968.10[1423.96-2341.68] pg/mL) than in the no-PRP group (2815.71[2440.70-3395.52] pg/mL) (*P* = 0.015). The control group had the lowest VEGF levels (582.70[380.28-924.23] pg/mL), which were statistically different from the two experimental groups (*P* = 0.008, *P* < 0.001). In contrast, although the CTGF concentration was higher in the PRP group (460.43[401.73-506.67] pg/mL) than in the no-PRP group (415.48[307.04-523.19] pg/mL), the difference was not statistically significant. In addition, there was a statistically significant difference in the CTGF/VEGF ratio between the PRP and no-PRP groups (*P* = 0.002), with the PRP group being significantly higher than the no-PRP group. Vitreous VEGF and CTGF levels are presented in Table 3 and Fig. 3.

**Table 3:** Vitreous VEGF and CTGF levels in three groups

	Study group			*P* value		
Cytokines	PRP group (*n* = 18)	No-PRP group (*n* = 18)	Control group (*n* = 10)	P_1_	P_2_	P_3_
VEGF (pg/mL)	1968.10(1423.96-2341.68)	2815.71(2440.70-3395.52)	582.70(380.28-924.23)	**0.015** ^**^**^	**0.008** ^**^**^	**< 0.001** ^**^**^
CTGF (pg/mL)	460.43(401.73-506.67)	415.48(307.04-523.19)	434.07(395.67–474.70)	0.601^^^		
CTGF/VEGF	0.27(0.13–0.34)	0.13(0.11–0.18)	-	**0.002** ^**~**^	-	-

VEGF: vascular endothelial growth factor; CTGF: connective tissue growth factor. ^^^Kruskal–Wallis test with post-hoc Bonferroni correction. ^~^Mann-Whitney U test. P1, P2, and P3 are the comparisons between the PRP and no-PRP groups, the PRP and control groups and the no-PRP and control groups, respectively

Multiple linear regression analyses were performed to isolate the independent association of PRP treatment with vitreous cytokine levels. After adjusting for potential confounders including diabetes duration, HbA1c, and PVD status, PRP treatment was independently associated with significantly lower VEGF levels (B=-926.559, 95%CI -1442.742 to -410.376; *P* < 0.001) and a higher CTGF/VEGF ratio (B = 0.14, 95%CI 0.05 to 0.23; *P* = 0.004) (Table 4).

**Table 4:** Multiple linear regression analyses of factors associated with vitreous VEGF levels and CTGF/VEGF ratio

Model and Predictors	Β Coefficient	95% CI for Β	*P* Value
**Dependent Variable: VEGF**			
Overall Model	F = 3.59, *P* = 0.017, R²=0.324		
PRP treatment (Yes)	-926.559	(-1442.742, -410.376)	**< 0.001**
Diabetes Duration (year)	1.603	(-29.613, 32.820)	0.917
HbA1c (%)	-15.387	(-238.544, 207.770)	0.889
PVD (no/partial)	-262.194	(-988.101, 463.713)	0.477
**Dependent Variable: CTGF/VEGF**			
Overall Model	F = 2.73, *P* = 0.048, R²=0.267		
PRP treatment (Yes)	0.140	(0.048, 0.233)	**0.004**
Diabetes Duration (year)	0.000	(-0.005, 0.006)	0.954
HbA1c (%)	-0.006	(-0.046, 0.034)	0.758
PVD (no/partial)	0.051	(-0.078, 0.181)	0.426

CI, confidence interval; VEGF, vascular endothelial growth factor; CTGF, connective tissue growth factor; PRP, panretinal photocoagulation; PVD, posterior vitreous detachment

Given the limited sample size in each time-based subgroup, the PRP group was subdivided into two cohorts according to the interval between PRP completion and vitreous sampling: ≤12 months group (*n* = 12) and > 12 months group (*n* = 6). No significant differences in VEGF levels, CTGF levels, or the CTGF/VEGF ratio were found between these subgroups (Table 5). A post hoc power analysis was performed for the primary outcome, vitreous VEGF levels. Using the observed effect size (Cohen’s d = 0.79) and an alpha of 0.05, the achieved power was only 31.7%. This indicates a high risk of Type II error, and the non-significant finding should be interpreted with caution, as the analysis was underpowered to detect clinically relevant differences between subgroups.

**Table 5:** Vitreous VEGF and CTGF levels in PRP subgroups by interval between PRP and vitreous sampling

	≤ 12 months (*n* = 12)	> 12 month (*n* = 6)	*P* value
VEGF (pg/mL)	2098.16(1577.52-2595.80)	1469.12(1206.45-1932.88)	0.134^~^
CTGF (pg/mL)	449.92(384.03-512.98)	461.67(382.61-505.28)	0.925^~^
CTGF/VEGF	0.23(0.13–0.32)	0.31(0.17–0.45)	0.303^~^

VEGF: vascular endothelial growth factor; CTGF: connective tissue growth factor. ^~^Mann-Whitney U test

**Fig. 4:**
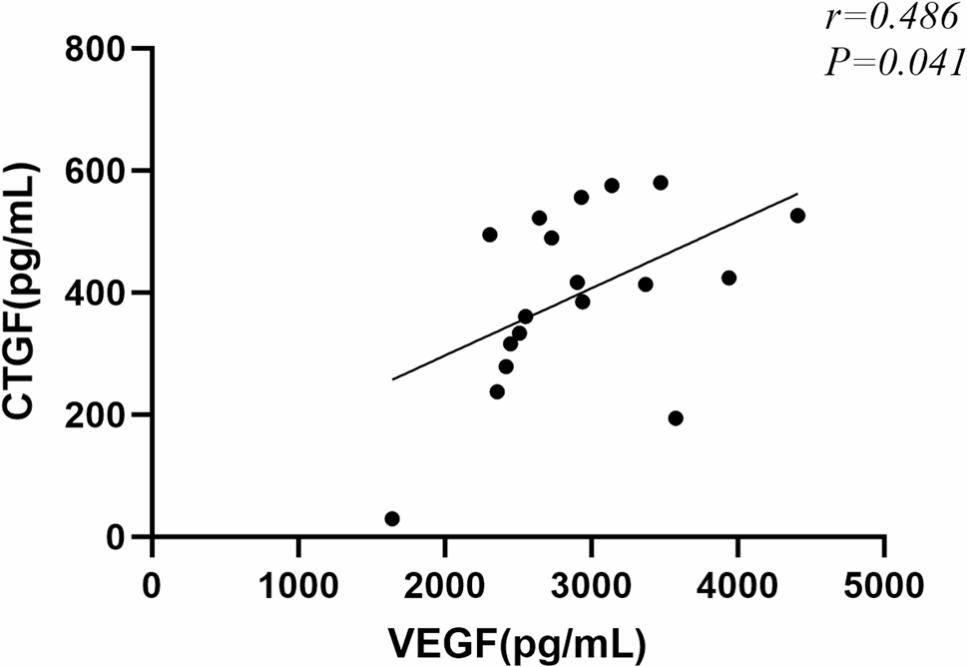
Correlation between the concentration of CTGF and VEGF in no-PRP group vitreous

**Fig. 5:**
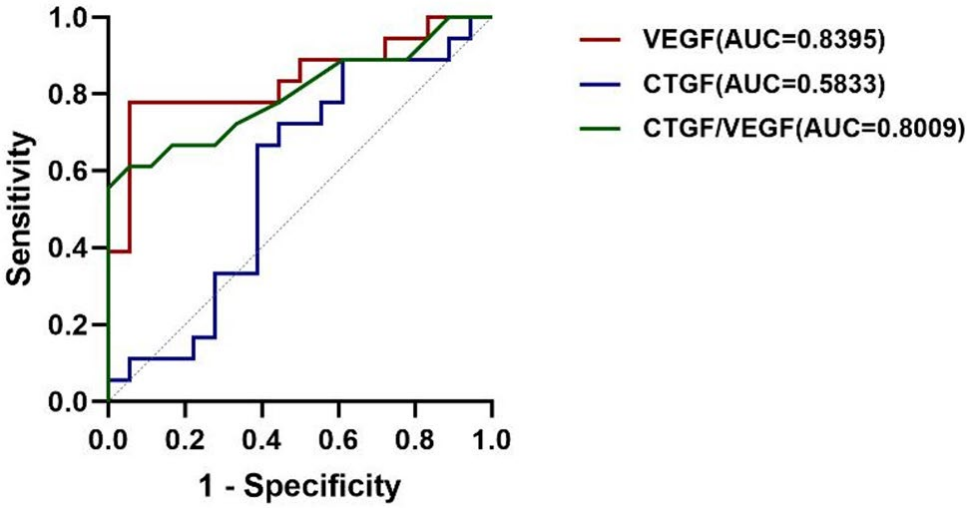
ROC curves for vitreous VEGF, CTGF and CTGF/VEGF ratio in PDR patients with or without prior PRP therapy

Further correlation analysis revealed a significant positive association between VEGF and CTGF levels in the no-PRP group with a Spearman’s ρ value of 0.486 (*P* = 0.041), but not in the PRP group (*P* = 0.627) (Fig. 4). None of the measured factors, including VEGF, CTGF levels, and CTGF/VEGF ratio, correlated with PVD type in either the no-PRP or PRP groups. ROC curve analysis determined the optimal cut-off values of VEGF and the CTGF/VEGF ratio for distinguishing the PRP and no-PRP groups (Table 6). VEGF had the largest AUC, whereas CTGF alone was not a significant discriminatory biomarker (Fig. 5).

**Table 6:** Sensitivity and specificity of vitreous VEGF, CTGF and CTGF/VEGF ratio as differentiating biomarkers of patients with PDR with and without previous PRP

Variable			Optimal cut off			AUC		Sensitivity (%)		Specificity (%)	*P* value
VEGF			2260.43 pg/mL			0.8395		94.4		77.8	< 0.001
CTGF			364.84/420.16 pg/mL			0.5833		88.9/72.2		38.9/55.6	0.39
CTGF/VEGF			0.2012			0.8009		61.1		94.4	0.002

VEGF: vascular endothelial growth factor; CTGF: connective tissue growth factor. AUC: area under the curve

### Intraoperative assessment of fibrosis-related findings

Although no extensive fibrous proliferation was observed in either group intraoperatively, the PRP group exhibited slightly dense, white fibrous membranes at the vitreoretinal adhesion sites, consistent with inactive or regressed neovascularization. In contrast, the adhesion sites in the no-PRP group were characterized by active neovascular complexes with a more brittle texture and a greater tendency to bleed.

## Discussion

In this cross-sectional study, we found that vitreous VEGF levels were significantly lower and the CTGF/VEGF ratio significantly higher in PRP-treated PDR patients compared to treatment-naïve patients. Our findings support the hypothesis that PRP treatment is associated with an altered relationship between VEGF and CTGF levels in PDR patients, suggesting a pro-fibrotic shift. Traction from microfibrous vascular membranes and the vitreous may represent key mechanisms contributing to VH after PRP treatment. However, the cross-sectional design only captures data at surgery and precludes causal inference. Therefore, our results demonstrate an association, rather than a causal relationship, between PRP and the observed cytokine alterations.

Angiogenesis and fibrosis are two important processes in the progression of PDR. Under normal intraocular conditions, a dynamic balance is maintained between angiogenic and anti-angiogenic factors. VEGF physiologically regulates the proliferation and assembly of endothelial cells, supporting vascular survival during angiogenesis. However, chronic hyperglycemia and hypoxia lead to VEGF overexpression, which promotes abnormal angiogenesis by inducing endothelial cell proliferation, migration, and increased vascular permeability [18]. Previous studies have consistently reported elevated VEGF levels in the vitreous and aqueous humor of patients with PDR and diabetic macular edema [19, 20]. CTGF plays an important role in driving the transition of PDR from the neovascular to the fibrotic phase, and its expression is correlated with the degree of fibrosis. Its expression is significantly increased in the vitreous and epiretinal membranes of patients with proliferative vitreoretinal disorders [21, 22]. The prevailing concept is that VEGF overexpression drives angiogenesis and CTGF production, which in turn suppresses VEGF. When the balance shifts to a certain threshold ratio, the angio-fibrotic switch is thought to occur; and the CTGF/VEGF ratio is the strongest predictor of the angio-fibrotic switch in PDR patients [5, 8, 9, 23].

PRP is essential for delaying the development of PDR. Studies have confirmed that it can promote neovascularization regression, reduce the incidence of VH, and maintain long-term visual and anatomical stability [15, 24, 25]. This may explain why the PRP group had a better baseline BCVA compared to the no-PRP group. Previous studies have confirmed that PRP treatment can effectively reduce VEGF levels in PDR patients [14, 19, 26]. In addition, Kuiper et al. predicted that there would be a transient increase in intraocular fibrosis after laser or anti-VEGF treatment, presenting as a transition from pre-retinal angiogenesis to the fibrosis progression phase [9]. In the context of diabetic macular edema, Guyer et al. found that subretinal fibrosis may be induced at three months after retinal laser treatment [27]. Li et al. found that CTGF promotes the fibrovascular reaction in murine retinal ischemia after laser injury [28]. However, changes in vitreous CTGF levels and CTGF/VEGF ratio after PRP treatment have not been investigated. In our study, we found that VEGF level was significantly decreased and CTGF/VEGF ratio was significantly increased in the PRP group compared to the no-PRP group, while CTGF level was not statistically different between the two experimental groups. Correlation analysis showed that VEGF and CTGF levels were strongly correlated before but not after PRP treatment. This distinctive pattern suggests that the alteration in the cytokine profile associated with PRP was primarily driven by the suppression of angiogenesis, while pro-fibrotic signaling might persist rather than be upregulated. This resulted in an elevated CTGF/VEGF ratio, indicating a shift in the molecular balance towards a relative pro-fibrotic microenvironment, rather than direct upregulation of fibrotic pathways by PRP. When the CTGF/VEGF ratio exceeds a specific threshold, it may mark or facilitate an angio-fibrotic switch. The high specificity (94.4%) of the CTGF/VEGF ratio cut-off value suggests that exceeding this threshold is highly indicative of prior PRP treatment and the associated pro-fibrotic state. This could serve as a valuable biomarker for risk stratification in the future. In addition, our intraoperative observations support the pro-fibrotic state, as PRP-treated eyes exhibited regressed neovascular membranes with more prominent microfibrous strands and firmer vitreoretinal adhesion.

Notably, intraocular VEGF and CTGF levels are influenced by many factors besides PRP. Zhao et al. confirmed that the CTGF/VEGF ratio appears to be higher in young PDR patients than in older ones [29]. Barbara et al. found that vitreous VEGF levels are lower in patients with well-controlled glucose, while blood glucose fluctuations and lipid abnormalities will induce VEGF synthesis [30]. Intravitreal injection of anti-VEGF therapy may exacerbate fibrosis risk in PDR patients by simultaneously reducing VEGF and elevating CTGF concentrations [31–33]. In addition, intraocular cytokine levels vary with time after laser treatment. Itaya et al. reported an increase in VEGF levels within one week after scatter photocoagulation. They observed that VEGF levels were highly elevated on the first day, peaked on the third day, and began to decline on the seventh day [34]. Photocoagulated RPE cells in vitro were also found to cause a transient upregulation of VEGF at 6 h, but by 72 h expression was reduced to pre-photocoagulation levels [35]. PRP appears to affect the intraocular cytokine levels for a longer time than expected. Kwon et al. confirmed that aqueous VEGF levels gradually decreased over time after PRP treatment, as they found patients who received PRP within 6 months exhibited higher levels of VEGF than those received PRP more than 12 months previously [19]. However, in our subgroup analysis based on the interval between the last PRP session and vitreous sampling, we found no significant differences in VEGF and CTGF levels. Due to the low statistical power, this negative finding should be interpreted with caution. Furthermore, the expression of VEGF is significantly influenced by hypoxia inducible factor-1 (HIF-1) and correlated with other angiogenesis or inflammation mediators such as syndecan-1, placental growth factor (PIGF), etc. [20]. In summary, the regulation and balance between VEGF and CTGF are complex processes influenced by numerous factors.

The reasons for the occurrence of VH after PRP treatment should be further investigated. Recently, a predictive model for worsening DR after PRP found that younger age, lower baseline BCVA, diabetic nephropathy, and hyperlipidemia were independent risk factors [36]. It is well established that persistent or recurrent neovascularization is associated with VH occurrence. Studies have shown that intensive initial PRP and additional adjunctive laser treatment may reduce the risk of VH [37]. Prophylactic and periodic intravitreal anti-VEGF injections (approximately 3–4 months intervals) are an option to prevent VH recurrence by facilitating neovascularization regression [38]. Furthermore, Baget-Bernaldiz et al. observed residual or recurrent fibrovascular membranes in most PDR patients with recurrent VH after initial PPV, suggesting that traction factor may play an important role in VH recurrence [39]. In our study, although all patients included were free of significant fibroproliferative membranes intraoperatively, we hypothesize that microfibrovascular membrane traction, potentially induced by the upregulated CTGF/VEGF ratio, may represent an additional key mechanism contributing to VH after PRP treatment. In addition, the status of PVD has been identified as one of the key factors influencing the development of PDR [39, 40]. Complete PVD is a strong negative risk factor for retinal neovascularization, whereas partial PVD or no PVD is a risk factor for retinal neovascularization by providing a scaffold for neovascular proliferation and migration [41]. Depending on the size of the vitreoretinal adhesion zone, PVD can induce various pathological events at the vitreoretinal interface including VH [42]. Baget-Bernaldiz et al. found that patients who suffered recurrent VH had a higher percentage of the posterior vitreous attached to the retina compared to patients with single VH [39]. In our study, no PVD was more prevalent in the PRP group, partial PVD was more prevalent in the no-PRP group, and complete PVD was less common in both groups.

The association between PRP and cytokine alterations must be considered alongside potential confounders. Although not statistically significant, the longer median duration of diabetes in the PRP group compared to the no-PRP group is clinically noteworthy, as it can influence the severity of retinal ischemia and cytokine expression. Poor glycemic control, as reflected by HbA1c, also exerts a significant influence on cytokine alterations [30]. Furthermore, the differential distribution of PVD types between groups represents another potential confounder given its established role in PDR progression [39]. Our multiple linear regression analysis was specifically adjusted for these factors. The persistence of a significant independent association between PRP and both lower VEGF levels and a higher CTGF/VEGF ratio after adjusting for diabetes duration, HbA1c, and PVD status provides more robust evidence for the specific effect of PRP treatment.

Separate from the comparisons within the PDR cohort, the demographic disparity between the control group and the PDR cohorts should be considered. The control group was predominantly female and slightly older than the PDR patients. Literature suggests that the CTGF/VEGF ratio may be lower in older PDR patients [29], indicating a potential confounding effect of age on our control data. In contrast, evidence for the substantial influence of sex on these cytokines is lacking. Therefore, while the control group provides a valuable non-diabetic reference, direct inter-group comparisons of absolute cytokine levels should be interpreted with caution. It is crucial to note that this potential limitation does not affect the primary study conclusions, which are robustly supported by the comparison between the well-matched PRP and no-PRP groups.

Several limitations of our study should be considered when interpreting the results. First, the cross-sectional nature of our design captures data at a single time point and precludes causal inference regarding the effect of PRP on cytokine dynamics. Second, although a two-step evaluation process was implemented to improve the accuracy of PVD classification, the assessment was limited by the presence of vitreous hemorrhage and vitreoschisis [43]. The interpretation of both US images and intraoperative findings remains subjective, and the lack of a formal assessment of inter-observer reliability means the potential for classification bias cannot be fully excluded. Third, the modest sample size challenges the robustness of some analyses, particularly the underpowered exploratory subgroup analyses, and increases the risk of overfitting in our multiple regression models. This may affect the generalizability of all findings. Finally, the influence of unmeasured confounders, such as renal function, lipid profile or systemic hypertension, cannot be ruled out. Future research should prioritize longitudinal designs with serial measurements before and after PRP in the same eyes to provide the most robust control for patient-specific confounders. Such large-scale prospective studies are strongly warranted to elucidate the precise temporal dynamics of VEGF, CTGF, and their ratio, ultimately defining the critical window for the angio-fibrotic switch and identifying patients at highest risk for fibrotic complications and VH.

## Conclusion

In this cohort, PRP treatment was associated with an altered relationship between VEGF and CTGF levels in PDR patients, characterized by an upregulation of the CTGF/VEGF ratio and suggestive of a pro-fibrotic shift. Our findings suggest that traction from microfibrous vascular membranes and vitreous body may represent key mechanisms contributing to VH in PDR patients after PRP treatment.

## Data Availability

All data generated or analyzed during this study are included in this published article. Further enquiries can be directed to the corresponding author.

## Abbreviations

PRP: Panretinal photocoagulation
VEGF: Vascular endothelial growth factor
CTGF: Connective tissue growth factor
VH: Vitreous hemorrhage
PPV: Pars plana vitrectomy
IMH: Idiopathic macular hole
DM: Diabetes mellitus
ELISA: Enzyme-linked immunosorbent assay test
PVC: Posterior vitreous cortex
PVD: Posterior vitreous detachment
BCVA: Best-corrected visual acuity
LogMAR: Logarithm of the minimum angle of resolution
US: B-scan ultrasonography
